# Computational modeling of visual salience alteration and its application to eye-movement data

**DOI:** 10.3389/fnins.2025.1614468

**Published:** 2025-08-13

**Authors:** Yoshihisa Fujita, Toshiya Murai, Jun Miyata

**Affiliations:** ^1^Department of Psychiatry, Graduate School of Medicine, Kyoto University, Kyoto, Japan; ^2^Department of Psychiatry, Aichi Medical University, Aichi, Japan

**Keywords:** visual salience, saliency map, computational model, neural network, eye movement

## Abstract

Computational saliency map models have facilitated quantitative investigations into how bottom-up visual salience influences attention. Two primary approaches to modeling salience computation exist: one focuses on functional approximation, while the other explores neurobiological implementation. The former provides sufficient performance for applying saliency map models to eye-movement data analysis, whereas the latter offers hypotheses on how neuronal abnormalities affect visual salience. In this study, we propose a novel saliency map model that integrates both approaches. It handles diverse image-derived features, as seen in functional approximation models, while implementing center-surround competition—the core process of salience computation—via an artificial neural network, akin to neurobiological models. We evaluated our model using an open eye-movement dataset and confirmed that its predictive performance is comparable to the conventional saliency map model used in eye-movement analysis. Beyond eye-movement prediction, our model enables neural-level simulations of how neurobiological disturbances influence salience computation. Simulations showed that parameter changes for excitatory-inhibitory balance, baseline neural activity, and synaptic connection density affected the contrast between salient and non-salient objects—in other words—the weighting of salience. Finally, we demonstrated the model’s potential for quantifying changes in salience weighting as reflected in eye movements, highlighting its ability to bridge both predictive and neurobiological perspectives. These results present a novel strategy for investigating mechanisms underlying abnormal visual salience.

## 1 Introduction

The brain receives vast amounts of information from sensory organs, including stimuli important for survival and those that can be ignored without consequence. To enable appropriate responses, attention must be selectively directed at each moment. Studies on the visual system suggest that salience, based on low-level visual features, guides bottom-up attention and influences eye movements (Veale et al., 2017). For example, if there is a red object among many blue objects, the red one stands out and attracts attention. Typical features that elicit such “pop-out” effects in static images include luminance, color, and orientation (Turatto and Galfano, 2000; Wolfe et al., 1992). Because natural scenes contain varying levels of luminance, diverse colors, and multiple orientations, salience in these contexts is determined by the combination of these features. To elucidate the complex process of salience computing, both theoretical and experimental approaches have been employed.

The saliency map has contributed to the quantitative investigation of visual salience. It is a two-dimensional map showing how perceptually conspicuous each region is in the corresponding visual stimulus. Koch and Ullman originally proposed the saliency map as a concept (Koch and Ullman, 1985), and its computational implementation was first reported by Itti et al. (1998). In their saliency map model, visual features such as orientation, color, and luminance are extracted from a visual stimulus and separately processed in various feature maps. Within each map, center-surround competition weakens the activity corresponding to abundant features in the visual stimulus and strengthens the activity associated with unique features. Feature maps are then integrated and finally output as a saliency map.

The saliency map model has been applied to eye-movement analysis and has enabled the exploration of the neural basis of visual salience (Veale et al., 2017). Although eye movements are influenced not only by bottom-up perception but also by top-down cognition, such as the semantic understanding of scenes and goal-related information, it was shown that fixations tend to cluster in highly salient regions indicated by the saliency map (Foulsham and Underwood, 2008; Itti, 2005). Therefore, the effect of bottom-up visual salience on eye movements can be evaluated using the saliency map. Eye-movement analysis of monkeys with brain lesions indicated that visual salience involves not only the primary visual cortex but also other brain areas (Yoshida et al., 2012). Electrophysiological recordings from the brains of monkeys during free viewing have shown a correlation between the saliency map and the activity of the midbrain structure known as the superior colliculus (White et al., 2017).

Researchers have also explored how neurons implement the center-surround competition, which is supposed to be the central process of visual salience computation. One account is that the competition is achieved through lateral interaction whereby closely located neurons enhance each other’s activity and distant neurons inhibit each other’s activity. It is based on a simple theoretical model of local competition in the visual system (Von Der Malsburg, 1973). Such lateral interaction has been supported by *in vitro* electrophysiological recordings of the superior colliculus in mouse brain slices (Phongphanphanee et al., 2014).

Recent studies have focused on abnormalities in visual salience associated with mental disorders. Patients with schizophrenia exhibit differences in eye movements compared to healthy controls, quantified as shorter scanpath length and impaired inhibition of return (Okada et al., 2021; Sprenger et al., 2013). Differences in visual salience have been observed by applying the saliency map model to analyze eye movements in patients (Yoshida et al., 2024). Another large-scale study found a relationship between affected visual salience and multiple mental disorders (Miura et al., 2025). As biological research has suggested various neurobiological abnormalities related to mental disorders, such as excitatory-inhibitory imbalance (Yizhar et al., 2011), disrupted signal-to-noise ratio (Jacob et al., 2013) and synaptic disconnectivity (Ellison-Wright and Bullmore, 2009; Garey et al., 1998), these abnormalities may affect visual salience and thereby cause symptoms. The neural basis of visual salience may also provide insights into the mechanisms underlying abnormal visual salience (Miyata, 2019). Because lateral interactions involving local excitatory connections and distant inhibitory connections are suggested to play a role in salience computing, its alteration may underlie abnormal visual salience.

A saliency map model that accounts for these abnormalities can facilitate biological investigations into the mechanisms of abnormal visual salience. However, the conventional saliency map model (Itti et al., 1998) used in eye-movement analyses has limitations in addressing this issue. This is mainly because the neural implementation of saliency computation is not sufficiently addressed in this model; in other words, center-surround competition is not implemented through artificial neural networks. A computational model that integrates neurobiological characteristics of center-surround competition should be used to tackle this problem.

Saliency map models based on artificial neural networks have been proposed in previous research (de Brecht and Saiki, 2006; Soltani and Koch, 2010). While they can capture biophysical aspects of neurons in visual salience computing, their simulations were performed only on very simple visual stimuli, such as rectangular bars on a black background. These models did not show sufficient ability to predict eye movements, which is necessary for application to eye-movement analysis. Recent machine learning techniques have enabled models based on convolutional neural networks to accurately predict human eye movements (Kroner et al., 2020). However, owing to end-to-end learning in these models, all processes influencing eye movements, including top-down cognition, may be reflected in the same artificial neural network. Therefore, these models face challenges in testing hypotheses that focus on local circuits of the brain responsible for specific information processing (i.e., center-surround competition).

In this study, we propose a new computational saliency map model based on de Brecht and Saiki (2006), which implements center-surround competition using artificial neural networks with excitatory center-inhibitory surround lateral connections. Their model has the advantage of incorporating neurobiological characteristics, but it can only process simple visual stimuli. Therefore, we extend their model to enable salience computation for complex visual stimuli such as natural images. The feature extraction algorithm was updated to detect complex features in natural images, and we searched for proper values of lateral connection weights to ensure that the output saliency map had sufficient performance in eye-movement prediction. To evaluate its performance in eye-movement prediction, we used an open dataset for eye movements (Borji and Itti, 2015). We compared the performance of different saliency map models in predicting eye movements for complex visual stimuli and confirmed that our model achieves comparable performance to the model by Itti et al. (1998), which has been applied to eye-movement analyses.

Using our proposed model, we introduced parameter changes to simulate neurobiological disturbances implicated in mental disorders and tested how the output saliency map was affected. Parameter changes related to excitatory-inhibitory imbalance in lateral interactions, disrupted signal-to-noise ratio, and reduced synaptic connections altered saliency computing such that the difference in salience between salient and non-salient objects changed. Finally, we tested our model as a tool for quantifying individual differences in the weighting of salience by parameter estimation from artificially generated fixation points.

## 2 Materials and methods

### 2.1 Visual stimulus and fixation data

**Figure 1:**
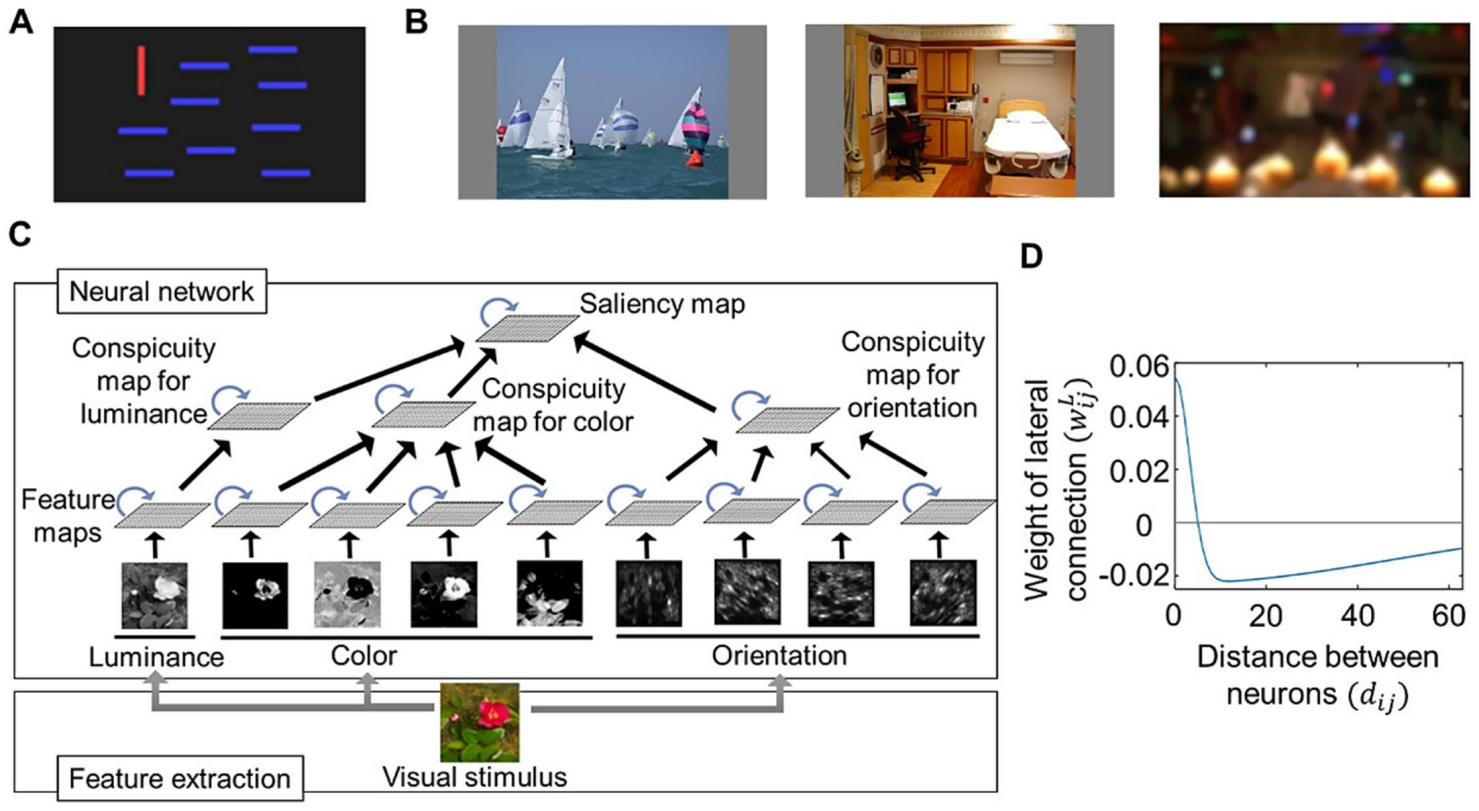
Architecture of computational modeling on visual salience. **(A)** An image used as a simple visual stimulus. **(B)** A few examples of images obtained from the CAT2000 dataset (Borji and Itti, 2015) and used as complex visual stimuli. Images included in “Action” (left), “Indoor” (middle), and “LowResolution” (right) categories. **(C)** Schematic diagram of our proposed computational saliency map model. Center-surround competition implemented by an artificial neural network (top) whose input was calculated through a feature extraction process (bottom). The neural network consists of multiple maps (quadrangles) implemented as two-dimensional arrays of neural populations. The neural populations within each map interact with each other via lateral connections (blue arrows) and receive signals from those in the lower layers via feedforward connections (black arrows). **(D)** Example distribution of lateral connection weights characterized by local excitatory and distant inhibitory connections. The weights are calculated by a difference-of-Gaussians function (Equation 17), where parameters $w_{I}$ and $\sigma_{L}$ are set as $w_{I}=140$ and $\sigma_{L}=3.2.$

We prepared a simple visual stimulus by creating an image with bars (Figure 1A) and used complex visual stimuli obtained from an open dataset by Borji and Itti (2015). This dataset includes various images used in eye-movement experiments, where subjects looked at each image for 5 s with no restrictions. There are 20 categories of images, including those representing natural images (e.g., Action, Indoor) as well as those with less meaningful content (e.g., Noisy, Low resolution); examples are shown in Figure 1B. For feature extraction, we used downsized images with resolutions of 384 × 216, 192 × 108, or 96 × 54 instead of the original 1920 × 1,080-pixel image. In addition, the RGB values of these images were normalized to a range of 0 and 1.

This dataset also includes fixation data collected during the experiments. We used the fixation data from 18 observers per image to evaluate our models’ ability to predict eye movements.

### 2.2 Model description

Figure 1C shows the schematic representation of our computational model. It consists of a feature extraction process and a saliency computing process, the latter of which is implemented using an artificial neural network. The extracted features provide input to the neural network, which performs center-surround competition and feature integration before outputting the saliency map. The basic structure follows the saliency map model proposed by de Brecht and Saiki (2006). While their model handles only features for two orientations and two colors, we expanded it to include features for four orientations (0, 45, 90, and 135 degrees), four colors (red, green, blue, and yellow), and luminance. In addition, we aligned the feature extraction algorithms for orientation and color similar with those used in the saliency map model by Itti et al. (1998), where orientation features are extracted by applying Gabor filters to images at multiple resolutions, and color features are extracted based on the opponency between red and green as well as blue and yellow.

The feature extraction algorithms reflect biological observations. Gabor filters resemble the response properties of orientation-selective neurons in the primary visual cortex (V1) (De Valois et al., 1982; Jain and Farrokhnia, 1991). The red-green and blue-yellow opponent-color responses are based on studies of the early visual system, demonstrating how cone inputs (L-, M-, and S-cones) are combined to allow fine-tuned chromatic discrimination (Engel et al., 1997).

The neural network consists of nine feature maps, each corresponding to a specific type of extracted feature. Each map processes signals for its respective feature separately. Within each map, neural activity competition is regulated through lateral interactions, characterized by local excitatory connections and distant inhibitory connections. The activity within the feature map layers is transmitted to the next layer, the conspicuity map layer, where signals are integrated into three conspicuity maps—orientation, color, and luminance. These maps undergo further competitive processing before their activity is ultimately combined into the saliency map.

As in the original Brecht’s model, neural activity was modeled using mean-field equations for neural populations with dynamic synapses (Tsodyks et al., 1998), rather than simulating individual neurons. This approach preserves neurobiological characteristics while reducing computational costs. As a result, we constructed the neural network with relatively low dimensionality, despite the fact that computing salience for natural images in the brain would require an enormous number of neurons. Many parameter values were retained from the original Brecht’s model, as they fall within physiologically realistic ranges. However, we adjusted the number of neural populations per map and connection weights, as these factors depend on the resolution at which the visual stimuli are processed.

The details of the model are described below, and it was implemented in MATLAB (version R2019b) with Image Processing Toolbox.

#### 2.2.1 Feature extraction

The luminance feature was calculated as the intensity of the image with a 96 × 54 resolution, as follows:


(1) $$F_{\mathrm{lum}}(x,y)=(V_{\mathrm{red}}(x,y)+V_{\text{green}}(x,y)+V_{\text{blue}}(x,y))/3,$$

where (x,y) represents the location of the image, and $V_{\mathrm{red}}(x,y)$, $V_{\text{green}}(x,y)$, and $V_{\text{blue}}(x,y)$ are the RGB values normalized to a range of 0 and 1.

Color feature extraction reflects the red-green and blue-yellow opponent-color responses in the visual system (Engel et al., 1997). The four features of red, green, blue, and yellow were extracted from the image with a 96 × 54 resolution, as follows:


(2) $$F_{\mathrm{col}}(\alpha ,x,y)=\{\begin{array}{cc} g(V_{\mathrm{red}}(x,y)-V_{\text{green}}(x,y))/F_{\mathrm{lum}}(x,y) & \alpha =1\\ g(V_{\text{green}}(x,y)-V_{\mathrm{red}}(x,y))/F_{\mathrm{lum}}(x,y) & \alpha =2\\ g(V_{\text{blue}}(x,y)-V_{\text{yellow}}(x,y))/F_{\mathrm{lum}}(x,y) & \alpha =3\\ g(V_{\text{yellow}}(x,y)-V_{\text{blue}}(x,y))/F_{\mathrm{lum}}(x,y) & \alpha =4, \end{array}$$

where α denotes the color index, $V_{\text{yellow}}(x,y)$ is defined as:


(3) $$V_{\text{yellow}}(x,y)=\min(V_{\mathrm{red}}(x,y),V_{\text{green}}(x,y)),$$

and g(x) is a linear gain function given by:


(4) $$g(x)=\{\begin{array}{cc} x & x>0\\ 0 & \text{otherwise}. \end{array}$$

Orientation features were extracted by applying Gabor filters that approximate the response of orientation-selective neurons in the primary visual cortex (Jain and Farrokhnia, 1991). We used the Gabor filter function given by:


(5) $$\begin{array}{c} G_{\theta }(x,y)=\exp(-\frac{x_{\theta }^{2}+{\left({\mathrm{\gamma y}}_{\theta }\right)}^{2}}{2\sigma^{2}})\exp(i\omega_{0}x_{\theta })\\ x_{\theta }=x\cos\theta +y\sin\theta \\ y_{\theta }=-x\sin\theta +y\cos\theta , \end{array}$$

where θ represents the orientation of the Gabor filters set as $\theta \in \{0^{{}^{\circ}},45^{{}^{\circ}},90^{{}^{\circ}},135^{{}^{\circ}}\}$, and γ, σ, and $\omega_{0}$ are filter parameters denoting the spatial aspect ratio, the standard deviation of the Gaussian kernel, and the frequency of the sinusoidal factor, respectively. We set the parameter values γ=0.5, σ=2, and $\omega_{0}=2$, following the implementation of the Itti-Koch model in the Graph-Based Visual Saliency (GBVS) toolbox for Matlab software (Harel et al., 2006). An image with a 96 × 54 resolution was convolved with Gabor filters, as follows:


(6) $$O_{\theta }(x,y)=\sum_{u}\sum_{v}G_{\theta }(x-u,y-v)V_{\mathrm{int}}(u,v)$$

where $V_{\mathrm{int}}(x,y)$ is the image intensity given by:


(7) $$V_{\mathrm{int}}(x,y)=(V_{\mathrm{red}}(x,y)+V_{\text{green}}(x,y)+V_{\text{blue}}(x,y))/3$$

Owing to the complex Gabor function defined in Equation 5, $O_{\theta }(x,y)$ has a complex value, and the orientation feature is calculated based on its absolute value. In addition, we applied Gabor filters to images at three different resolutions and averaged the resulting filter responses, as applying Gabor filters only to 96 × 54 images may capture only coarse-grained features. Therefore, the orientation feature is given by:


(8) $$F_{\mathrm{ori}}(\theta ,x,y)=\frac{\left(|O_{\theta }(x,y)|+|O_{\theta }^{\prime }(x,y)|+|O_{\theta }^{\prime \prime }(x,y)|\right)}{3}$$

where $O_{\theta }^{\prime }(x,y)$ and $O_{\theta }^{\prime \prime }(x,y)$ represent the values obtained by convolving the images with 192 × 108 and 384 × 216 resolutions, respectively, with the Gabor filters, and downsizing them to 96 × 54 resolutions. Fine-grained features can be extracted from 192 × 108 and 384 × 216 images and incorporated into the 96 × 54 orientation feature image. When Gabor filters are applied to 384 × 216 or 192 × 108 images, the extracted features span multiple pixels, enabling them to be preserved after downsampling to a resolution of 96 × 54.

#### 2.2.2 Neural network

The artificial neural network comprises a feature map layer, conspicuity map layer, and saliency map layer. The feature map layer has nine feature maps corresponding to nine features extracted from the image (luminance, four colors [red, green, blue, and yellow], and four orientations [0, 45, 90, and 135 degrees]). The conspicuity map layer has three conspicuity maps corresponding to luminance, color, and orientation features. The saliency map layer has a saliency map as the output. Each map was implemented as a 96 × 54 array of neural populations. Based on Equations 1–8 the input $I_{m,i}$ for neural population i in feature map m is given by:


(9) $$I_{m,i}=\{\begin{array}{cc} C_{\mathrm{lum}}F_{\mathrm{lum}}(x_{i},y_{i}) & m=1\\ C_{\mathrm{col}}F_{\mathrm{col}}(m-1,x_{i},y_{i}) & m=2,3,4,5\\ C_{\mathrm{ori}}F_{\mathrm{ori}}(45(m-6),x_{i},y_{i}) & m=6,7,8,9 \end{array}$$

where $\left(x_{i},y_{i}\right)$ denotes the coordinates of the neural population i, and $C_{\mathrm{lum}}(=30)$, $C_{\mathrm{col}}(=15)$, and $C_{\mathrm{ori}}(=20)$ are constants that cause neural activity within a reasonable range.

Each neural population receives signals from populations in the same map via lateral connections and signals from populations in the lower layer via feedforward connections. The activity $A_{m,i}(t)$ for neural population i in map m at time t was updated depending on these signals:


(10) $$\tau \frac{dA_{m,i}}{\mathrm{dT}}=-A_{m,i}(t)+b+0.5g(S_{L}(m,i,t)+S_{F}^{\text{Post}}(m,i,t)),$$

where $S_{L}(m,i,t)$ represents signals via lateral connections; $S_{F}^{\text{Post}}(m,i,t)$, feedforward connections; τ, time constant set to 0.03 s (30 ms); and g(x), linear gain function defined in Equation 4. Herein, we introduced b as the baseline activity, allowing the neural population to be active even in the absence of signals from other populations. We set b=0 for most simulations and b>0 for other simulations, assuming an abnormality in the neuronal signal-to-noise ratio. Because we consider t as time in milliseconds and $\frac{dA_{m,i}}{\mathrm{dT}}$ as the change in $A_{m,i}(t)$ per second, $A_{m,i}(t+1)$ is given by:


(11) $$A_{m,i}(t+1)=A_{m,i}(t)+0.001\frac{dA_{m,i}}{\mathrm{dT}}.$$

The feedforward signal $S_{F}^{\text{Post}}(m,i,t)$ depends on the connection between the maps. In our model, feature maps (m=1,…,9) received the input $I_{m,i}$ as defined in Equation 9; the conspicuity maps for luminance, color, and orientation (m=10,11,12, respectively) received signals from feature maps for luminance (m=1), color (m=2,3,4,5), and orientation (m=6,7,8,9); and the saliency map (m=13) received signals from the three conspicuity maps:


(12) $$S_{F}^{\text{Post}}(m,i,t)=\{\begin{array}{cc} I_{m,i} & m=1,\ldots ,9\\ \sum_{p=1}^{1}S_{F}^{\mathrm{Pre}}(p,i,t) & m=10\\ \sum_{p=2}^{5}S_{F}^{\mathrm{Pre}}(p,i,t) & m=11\\ \sum_{p=6}^{9}S_{F}^{\mathrm{Pre}}(p,i,t) & m=12\\ \sum_{p=10}^{12}S_{F}^{\mathrm{Pre}}(p,i,t) & m=13 \end{array}$$

where $S_{F}^{\mathrm{Pre}}(p,i,t)$ represents the signal from map p at the presynaptic layer to neural population i at the postsynaptic layer.

Synaptic transmission between each neural population is modeled based on the equations for dynamic synapses (Tsodyks et al., 1998), which describe the synaptic resources used by activated neurons that recover gradually over time. The lateral signals $S_{L}(m,i,t)$ and feedforward signals $S_{F}^{\mathrm{Pre}}(p,i,t)$ are expressed as follows:


(13) $$\begin{array}{c} S_{L}(m,i,t)=U_{\mathrm{SE}}^{L}\sum_{j}w_{\mathrm{ij}}^{L}z_{m,j}^{L}(t)A_{m,j}(t)\\ S_{F}^{\mathrm{Pre}}(p,i,t)=U_{\mathrm{SE}}^{F}\sum_{k}w_{\mathrm{ik}}^{F}z_{p,k}^{F}(t)A_{p,k}(t), \end{array}$$

where $U_{\mathrm{SE}}^{L}$ (=0.5) and $U_{\mathrm{SE}}^{F}$ (=0.5) are the utilization of synaptic efficacy parameters that denote the fractions of synaptic resources activated by an action potential; $w_{\mathrm{ij}}^{L}$ and $w_{\mathrm{ik}}^{F}$ are the weights of the lateral connections and feedforward connections, respectively; and $z_{m,j}^{L}(t)$ and $z_{p,k}^{F}(t)$ are the depressing effects of dynamic synapses for lateral connections and feedforward connections, respectively, which represent the fractions of available synaptic resources and are updated as follows:


(14) $$\begin{array}{c} \frac{dz_{m,j}^{L}}{\mathrm{dT}}=-U_{\mathrm{SE}}^{L}A_{m,j}(t)z_{m,j}^{L}(t)+\frac{1-z_{m,j}^{L}(t)}{\tau_{\mathrm{rec}}^{L}}\\ \frac{dz_{p,k}^{F}}{\mathrm{dT}}=-U_{\mathrm{SE}}^{F}A_{p,k}(t)z_{p,k}^{F}(t)+\frac{1-z_{p,k}^{F}(t)}{\tau_{\mathrm{rec}}^{F}}, \end{array}$$

where $\tau_{\mathrm{rec}}^{L}$ (=0.1 sec) and $\tau_{\mathrm{rec}}^{F}$ (=0.05 sec) are time constants that govern the rate of recovery of synaptic resources. As in Equation 11, $z_{m,j}^{L}(t+1)$ and $z_{p,k}^{F}(t+1)$ are given by:


(15) $$\begin{array}{c} z_{m,j}^{L}(t+1)=z_{m,j}^{L}(t)+0.001\frac{dz_{m,j}^{L}}{\mathrm{dT}}\\ z_{p,k}^{F}(t+1)=z_{p,k}^{F}(t)+0.001\frac{dz_{p,k}^{F}}{\mathrm{dT}} \end{array}$$

These depressing effects cause a temporary decrease in the strength of synaptic transmission if the neural populations are highly active, which contributes to the normalization of neural activity. We used the same values for parameters τ, $U_{\mathrm{SE}}^{L}$, $U_{\mathrm{SE}}^{F}$, $\tau_{\mathrm{rec}}^{L}$, and $\tau_{\mathrm{rec}}^{F}$ as those in the Brecht–Saiki model (de Brecht and Saiki, 2006), as they are within a physiologically realistic range (Markram et al., 1998; Tsodyks et al., 1998).

The connection weights for the feedforward and lateral connections $w_{\mathrm{ik}}^{F}$ and $w_{\mathrm{ij}}^{L}$ depend on the locations of the presynaptic and postsynaptic neural populations within the maps. The feedforward connection weights $w_{\mathrm{ik}}^{F}$ were set as follows:


(16) $$\begin{array}{c} w_{\mathrm{ik}}^{F}=\hat{w}(x_{i}-x_{k})\hat{w}(y_{i}-y_{k})\\ \hat{w}(x)=\{\begin{array}{cc} 0.14 & x=-2,2\\ 0.71 & x=-1,1\\ 1.13 & x=0 \end{array}, \end{array}$$

where $\left(x_{i},y_{i}\right)$ and $\left(x_{k},y_{k}\right)$ denote the coordinates of neural population i in the postsynaptic map and neural population k in the presynaptic map, respectively. In this setting, excitatory feedforward connections exist if the coordinates $\left(x_{i},y_{i}\right)$ and $\left(x_{k},y_{k}\right)$ are in close proximity.

The lateral connection weights $w_{\mathrm{ij}}^{L}$ were defined by a difference-of-Gaussian function to implement local excitatory connections and distant inhibitory connections (Figure 1D):


(17) $$w_{\mathrm{ij}}^{L}=\frac{w_{E}}{2\pi \sigma_{L}^{2}}\exp[\frac{-d_{\mathrm{ij}}^{2}}{2\sigma_{L}^{2}}]-\frac{w_{I}}{2\pi {\left(\beta \sigma_{L}\right)}^{2}}\exp[\frac{-d_{\mathrm{ij}}^{2}}{2{\left(\beta \sigma_{L}\right)}^{2}}],$$

in which $w_{E}$ and $w_{I}$ determine the amplitudes of the Gaussian components; $\sigma_{L}$ and β determine the standard deviations of the Gaussian components; and $d_{\mathrm{ij}}$ is the distance between the two neural populations i and j given by:


(18) $$d_{\mathrm{ij}}=\sqrt{{\left(x_{i}-x_{j}\right)}^{2}+{\left(y_{i}-y_{j}\right)}^{2}}.$$

The lateral connections enabled center-surround competition for salience computing, but the optimal values for $w_{E}$, $w_{I}$, $\sigma_{L}$, and β depend on the resolution at which the visual stimuli are processed. In addition, we assumed that there were individual differences in lateral interactions owing to variations in neurobiological characteristics. Therefore, we tested our model using various lateral connections by varying the values of $w_{I}$ and $\sigma_{L}$. We fixed $w_{E}=2.0$ for simplicity and β=15 to maintain the excitatory-center, inhibitory-surround structure.

### 2.3 Models for comparison

We used two additional models—the Brecht-Saiki model (de Brecht and Saiki, 2006) and the Itti-Koch model (Itti et al., 1998)—to compare eye-movement prediction performance with our proposed model. The Brecht-Saiki model was implemented with slight modifications to accommodate differences in visual stimuli while preserving its fundamental structure. This model processes two color features (red and green) and two orientation features (0 and 90 degrees). The color features are derived directly from RGB values, while the orientation features are extracted using Gabor filters applied to images with a 96 × 54 resolution. These features serve as inputs to the artificial neural network. Therefore, input $\overset{ˇ}{I_{m,i}}$ for neural population i in feature map m is represented as:


(19) $$\overset{ˇ}{I_{m,i}}=\{\begin{array}{cc} \overset{ˇ}{C_{\mathrm{col}}}V_{\mathrm{red}}(x_{i},y_{i}) & m=1\\ \overset{ˇ}{C_{\mathrm{col}}}V_{\text{green}}(x_{i},y_{i}) & m=2\\ \overset{ˇ}{C_{\mathrm{ori}}}|O_{0}(x_{i},y_{i})| & m=3\\ \overset{ˇ}{C_{\mathrm{ori}}}|O_{90}(x_{i},y_{i})| & m=4 \end{array},$$

where the constants $\overset{ˇ}{C_{\mathrm{col}}}$ (=30) and $\overset{ˇ}{C_{\mathrm{ori}}}$ (=30) are set to cause neural activity within a reasonable range. The neural network consists of maps, each of which is implemented as a 96 × 54 array of neural populations. The main differences in the neural network implementation from our proposed model (Equations 10–18) are the number of the maps and connections between them that underlie the feedforward signals. It has four feature maps (m=1,2,3,4), two conspicuity maps for color and orientation (m=5,6, respectively), and a saliency map (m=7). The feedforward signals received by neural population i in map m at time t are given by:


(20) $$\overset{ˇ}{S_{F}^{\text{Post}}}(m,i,t)=\{\begin{array}{cc} \overset{ˇ}{I_{m,i}} & m=1,\ldots ,4\\ \sum_{p=1}^{2}S_{F}^{\mathrm{Pre}}(p,i,t) & m=5\\ \sum_{p=3}^{4}S_{F}^{\mathrm{Pre}}(p,i,t) & m=6\\ \sum_{p=5}^{6}S_{F}^{\mathrm{Pre}}(p,i,t) & m=7 \end{array},$$

instead of Equation 12. Apart from the input (Equation 19) and feedforward signals (Equation 20), the other difference from the proposed model is that the lateral connection weights were set to be the same as those in the original Brecht-Saiki model. They are determined by Equation 17 with $w_{E}=5.0$, $w_{I}=250$, $\sigma_{L}=3.2$, and β=15 for feature maps and conspicuity maps, and there are no lateral connections in the saliency map.

We used the Itti-Koch model implemented in the GBVS toolbox for Matlab software (Harel et al., 2006). It computes salience based on a luminance feature, color features for red-green opponency and blue–yellow opponency, and orientation features for 0, 45, 90, and 135 degrees extracted using Gabor filters. The features were extracted from images at multiple scales (Gaussian pyramids) obtained from a visual stimulus. The center-surround competition was implemented without artificial neural networks. Instead, it followed these steps: first, normalization: each feature image was normalized to a fixed range $\left[0\ldots M_{g}\right]$; second, local maxima averaging: the average $\bar{M_{l}}$ of local maxima was computed, excluding global maximum $M_{g}$; third, global scaling: the entire map was multiplied by ${\left(M_{g}-\bar{M_{l}}\right)}^{2}$. After the center-surround competition for each feature, the feature images were integrated into the saliency map.

### 2.4 Evaluation of the model performance in eye-movement prediction

We evaluated the performance of our proposed model with various lateral connections, alongside the Brecht-Saiki and Itti-Koch models, by applying them to eye-movement data. Performance was evaluated by quantifying the correspondence between the saliency map and fixation points (ground truth) obtained from eye-movement measurements. We used the Normalized Scanpath Saliency (NSS) metric for evaluation (Bylinskii et al., 2019; Kümmerer et al., 2018). To compute NSS, the saliency map was normalized to have zero mean and unit variance, and the normalized saliency values at fixation locations were averaged. An NSS value greater than 0 indicated correspondence between the saliency map and fixation points, with higher values suggesting better eye-movement prediction. We used visual stimuli and fixation data from the CAT2000 dataset (Borji and Itti, 2015). Considering the computational cost of simulating our proposed model with various lateral connection parameters, we selected five random visual stimuli from each of the 20 categories (100 stimuli in total) and corresponding fixation data instead of using the entire dataset. The saliency map at steady state (timepoint t=400) was used for NSS metrics.

Here, we did not reproduce eye movement trajectories from the saliency map; instead, we simply used the saliency map as a reference metric for assessing the spatial distribution of fixation points. Predicting gaze trajectories requires incorporating additional characteristics into the model—such as saccade length, fixation duration, and inhibition of return—which increases model complexity. By using the NSS metric, we did not consider the individual sequence or path of eye movements, which offers a more tractable, though less precise, means of evaluating model performance.

### 2.5 Testing parameter estimation from simulated data

To assess our model’s ability to quantify individual differences in eye movements, we performed parameter estimation using artificially generated fixation points. This approach follows the parameter recovery method, which evaluates whether a computational model can be effectively applied to behavioral data analysis (Wilson and Collins, 2019). The testing process consisted of the following steps. First, fixation points were artificially generated based on the saliency map computed by our model using a predefined parameter value. Second, the model was used to compute saliency maps for the same visual stimulus with various parameter values. Third, the NSS metric was used to quantify the correspondence between the generated fixation points and the computed saliency maps. Ideally, the best correspondence (highest NSS score) should occur when the estimation parameter matches the value used to generate fixations. We tested the estimation of $w_{I}$, which determines the strength of lateral connections (as described in Equation 17), while keeping $\sigma_{L}$ fixed at 6.4. Fixation points were generated under the assumption that the normalized saliency map represents their probability distribution. Therefore, the saliency map was normalized such that its sum equaled 1, and fixation points were sampled accordingly. For each visual stimulus and parameter setting, 20 fixation points were generated, reflecting the scale of real experimental data used in saliency analysis. We used the same 100 CAT2000 visual stimuli from the model performance evaluation. The average NSS score across these 100 stimuli was computed, using the steady-state saliency map (timepoint t=400) for both fixation generation and NSS evaluation.

## 3 Results

**Figure 2:**
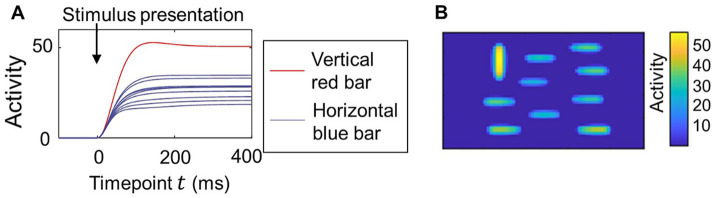
Transition of the neural activity computed in the proposed model for a simple visual stimulus. **(A)** Time course of the neural activity in the saliency map. Image of a vertical red bar and nine horizontal blue bars shown in Figure 1A used for the visual stimulus. The parameters for the lateral connections $w_{I}$ and $\sigma_{L}$ were set as $w_{I}=140$ and $\sigma_{L}=3.2,$ as in Figure 1D. The activity of neural populations in the region corresponding to each bar was averaged. **(B)** The activity of all neural populations in the saliency map at timepoint t=400 in the same simulation as that of **(A)**.

Our model computes the time course of neural population activity. Figure 2A shows an example of the time series of activity in the saliency map caused by the simple visual stimulus shown in Figure 1A, which includes a vertical red bar as a salient object and many horizontal blue bars as non-salient objects. The activity of neural populations increased after stimulus presentation and then reached a steady state. The activity corresponding to the red bar became larger than that corresponding to the blue bars, indicating that the center-surround competition weakened the activity for non-salient objects. There is still substantial activity for the blue bars (non-salient objects) at the steady state compared to the background (Figure 1B).

**Figure 3:**
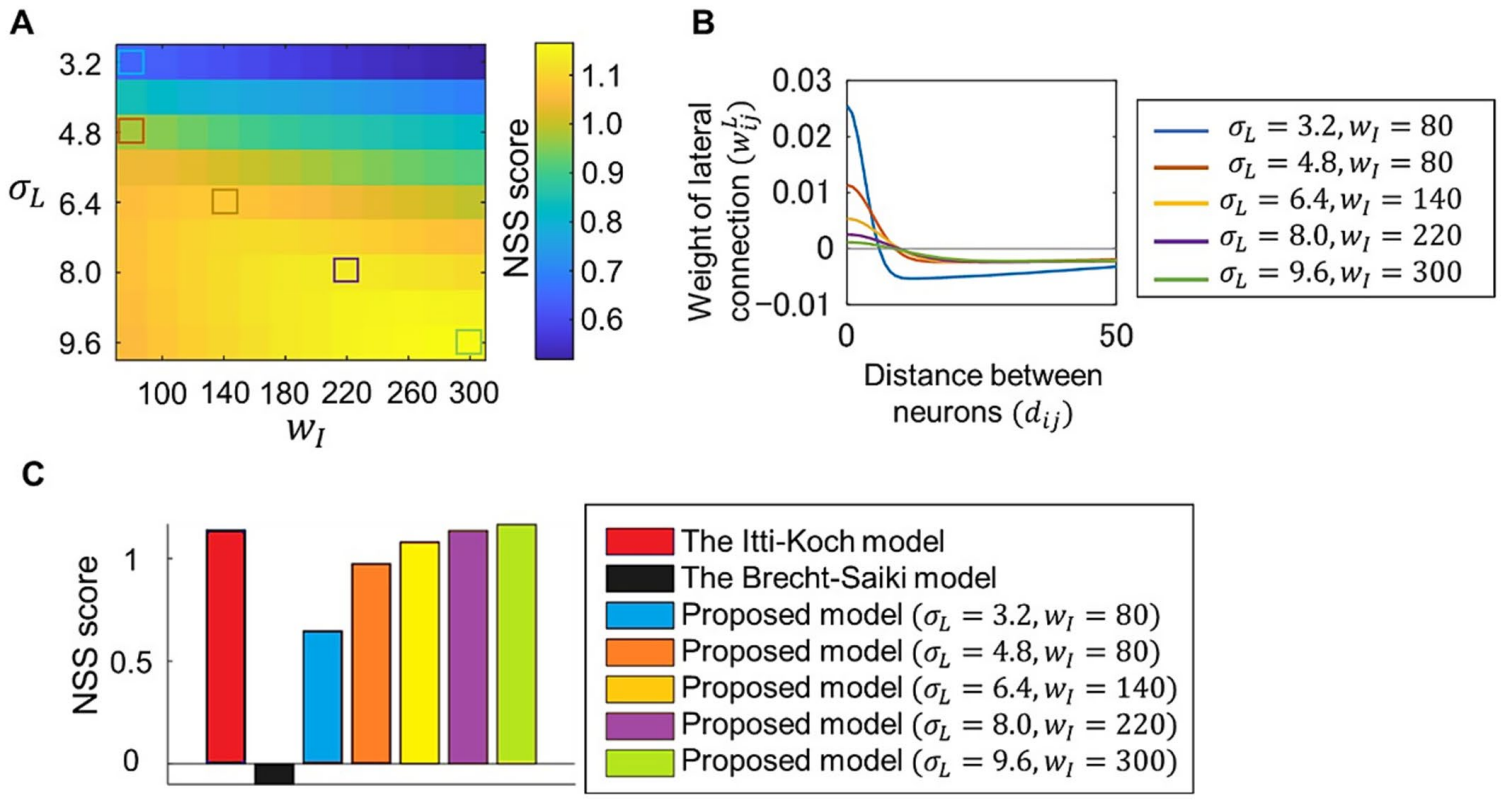
Evaluation of model performance in eye-movement prediction. **(A)** Correspondence between fixation data measured in experiments and the saliency map computed by the proposed model, in which various parameter values $w_{I}$ and $\sigma_{L}$ for lateral connections were set. Visual stimuli and fixation data included in CAT2000 dataset was used. Correspondence between fixations and the saliency map for each visual stimulus was calculated using NSS metrics, and the average NSS scores for 100 visual stimuli are plotted. **(B)** Distribution of lateral connection weights determined by five sets of parameter values corresponding to those denoted by colored squares in **(A)**. **(C)** Comparison of performances of different models. Performances are quantified utilizing NSS metrics as in **(A)**. Performances for the Itti-Koch model, the Brecht-Saiki model, and our proposed model with the five parameter sets denoted by colored squared in **(A)** are plotted.

We evaluated the ability of our model to predict eye movements using visual stimuli and fixation data included in the CAT2000 dataset (Borji and Itti, 2015). The correspondence between the saliency map and the fixation points for each visual stimulus was measured using the NSS metric, and the model’s performance in eye-movement prediction was quantified as the average NSS score for 100 visual stimuli. We tested the model with various values of parameters $w_{I}$ and $\sigma_{L}$, which determine the lateral connection weights as described in Equation 17 in the Methods section. Figure 3A shows that the model’s performance in predicting eye movements varied depending on these parameter values. The distribution of lateral connection weights corresponding to some of these parameter values is plotted in Figure 3B. Lateral connections with excessive excitatory or inhibitory connections showed relatively low performance. We then compared the performance of our model with two other models. One is the Itti-Koch model (Itti et al., 1998), which has been used for eye-movement analyses (Foulsham and Underwood, 2008; Miura et al., 2025; Yoshida et al., 2024) but does not address the neural implementation of center-surround competition. Our model, when using parameter values that provide balanced lateral connections, achieved comparable performance with the Itti-Koch model (Figure 3C). The second model is the Brecht-Saiki model (de Brecht and Saiki, 2006), which implements center-surround competition using an artificial neural network but is limited to simple features. The average NSS score for this model was close to zero, indicating that its ability to predict eye movements for complex visual stimuli was nearly at chance level.

**Figure 4:**
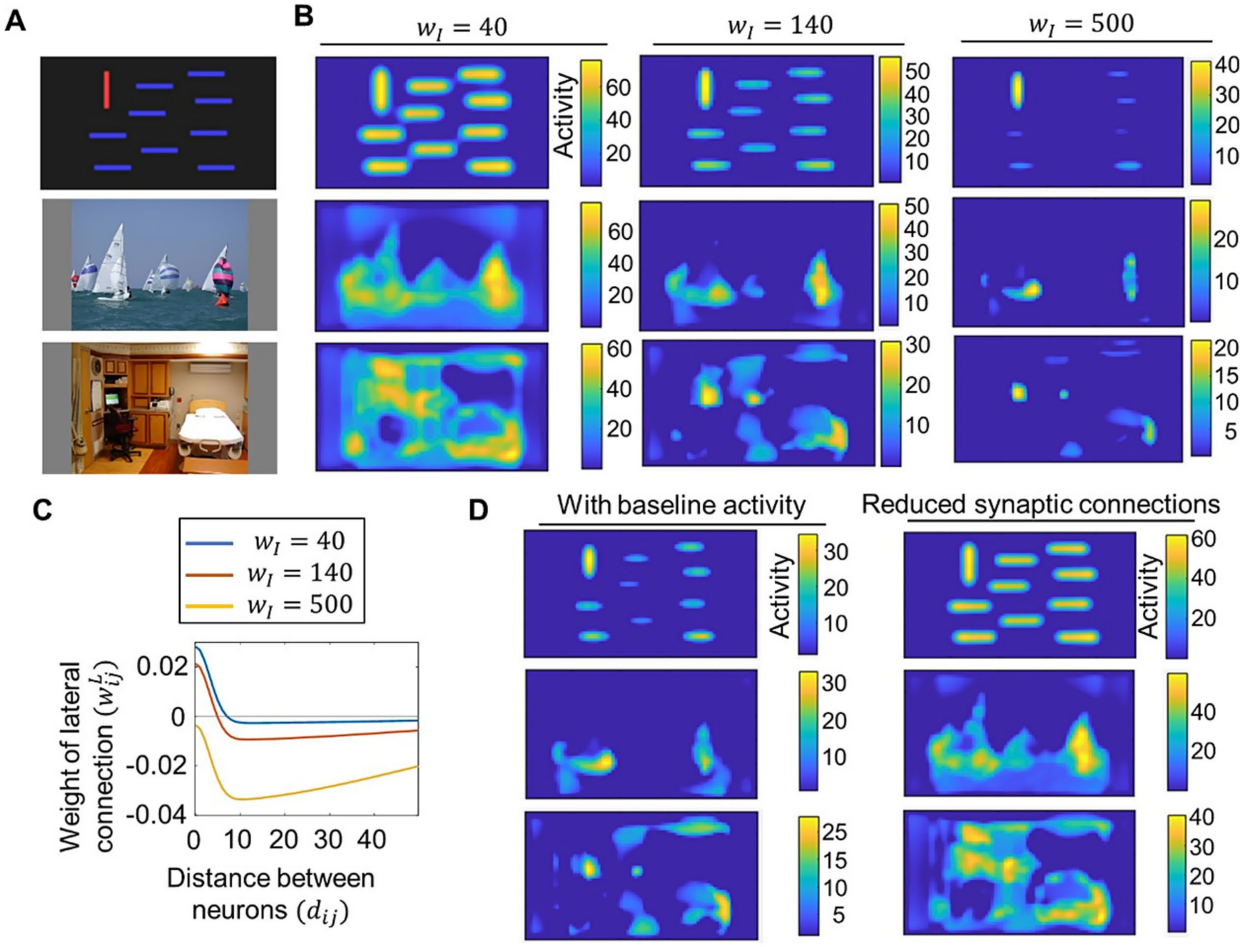
Demonstration of how saliency computing is altered by parameter changes in the proposed model. **(A)** Visual stimuli of bars (top), sailing (middle), and a room (bottom) used for the test. The latter two stimuli were obtained from CAT2000 dataset (Borji and Itti, 2015). **(B)** Saliency maps computed by the proposed model with varied values of the parameter $w_{I}$ that determined lateral connections. Neural activity of saliency maps at the steady state (timepoint t=400) for visual stimuli of bars (top row), sailing (middle row), and a room (bottom row). **(C)** Distribution of lateral connections used for computing saliency maps in **(B)**. **(D)** Saliency maps computed by the proposed model with baseline neural activity (left column) and with reduced synaptic connections (right column). Neural activity of saliency maps at the steady state (timepoint t=400) for visual stimuli of bars (top row), sailing (middle row), and a room (bottom row).

We next examined how saliency computation is altered by changes in the neural network parameters of our proposed model. We tested lateral connections with different excitatory-inhibitory balances and compared the resulting saliency maps. For clarity, we set $w_{I}$ as $w_{I}\in \{40,140,500\}$ and $\sigma_{L}$ fixed at 3.2 to construct lateral connections, which differ from the parameter settings used for model performance evaluation in Figure 3. Figures 4A,B show some examples of visual stimuli and the corresponding output saliency maps, while Figure 4C shows the distribution of lateral connections used in these cases. When inhibitory connections were reduced, our model computed similar saliency values for both salient objects and non-salient objects. In contrast, when inhibitory connections are increased, the saliency of non-salient objects became significantly weaker. These results indicate that the difference in saliency between salient and non-salient objects is influenced by the excitatory-inhibitory balance. We also tested parameter changes related to the signal-to-noise ratio and reduced synaptic connections. We introduced baseline activity for neural populations by setting the parameter b in Equation 10 to b=1 (b=0 for other simulations), allowing each neural population to exhibit weak activity even in the absence of signals from other neural populations. This adjustment, which can be regarded as modifying the signal-to-noise ratio of neural responses, resulted in a larger difference in saliency between salient and non-salient objects (Figure 4D, left). For reduced synaptic connections, we set the connection weights of 60% of randomly chosen lateral connections to 0, which increased saliency values for non-salient objects (Figure 4D, right).

**Figure 5:**
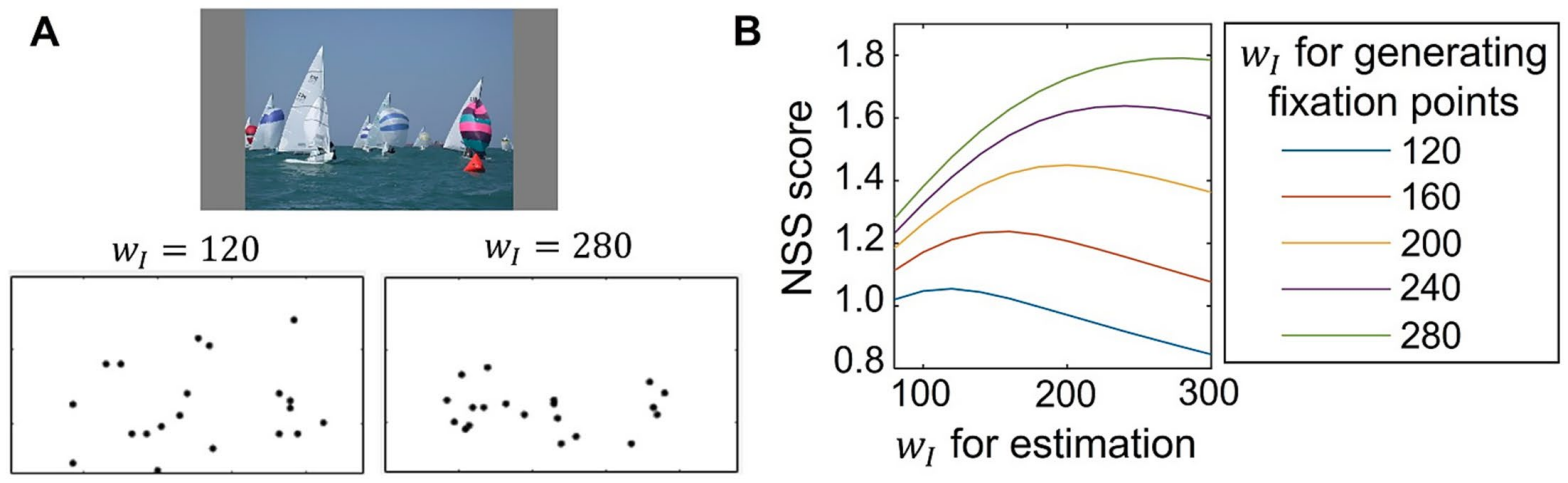
Test of parameter estimation from generated fixations. **(A)** Example of artificially generated fixation points for a visual stimulus (top) obtained from CAT2000 dataset (Borji and Itti, 2015). Fixation points were generated from the saliency map computed by the proposed model, where the parameter $w_{I},$ which modulates lateral connections, was set to 120 (bottom left) or 280 (bottom right). **(B)** Estimation of the parameter $w_{I}$ using fixation points generated artificially from the saliency map of the proposed model with varied values of $w_{I}\in \{120,160,200,240,280\}.$ The NSS metric was used to quantify the correspondence between the generated fixation points and the saliency map computed by the proposed model with various values of $w_{I}$ for estimation. The NSS scores, averaged for 100 visual stimuli, are plotted.

The results described above suggest that individual differences in saliency computation could be quantified by applying our model to eye-movement data analysis. To evaluate whether our model could function as a tool for analyzing eye-movement data, we tested parameter estimation by comparing the computed saliency map with artificial fixation data. The analysis was based on estimating the parameter value that provided the best correspondence between the computed saliency map and eye-movement data. Here, we used fixation data artificially generated from the saliency map computed by our model with specific parameters, which allowed us to directly compare the results of the parameter estimation with the true parameter value (i.e., the value used for generating data). The artificial fixation data was generated based on our model using five different settings where the parameters $w_{I}$, which determine lateral connection weights, were set as $w_{I}\in \{120,160,200,240,280\}$ (See Section 2.5 for more detailed methods). Representative examples of generated fixation points are shown in Figure 5A. When $w_{I}$ was set to the higher value, the model computed the saliency map in which salience for non-salient objects was weakened as shown in Figure 4B. Consequently, the generated fixations tended to cluster in regions of high salience. We then calculated NSS scores to evaluate the correspondence between the artificially generated fixation points and the saliency map computed by our model using various values of $w_{I}$ for estimation. The average NSS scores for 100 visual stimuli are plotted in Figure 5B, showing that NSS scores were highest when the estimated $w_{I}$ value for parameter estimation was close to the one used for generating the fixation data. This distribution is desirable because the estimated parameter value, which achieves the highest correspondence (i.e., the highest NSS score), should ideally match the true value used to generate the fixation data. These findings demonstrate our model’s potential for accurate parameter estimation from eye-movement data.

## 4 Discussion

In this study, we proposed a new computational model of abnormal bottom-up visual salience to facilitate the investigation of how neurobiological abnormalities alter salience computation. We designed the model to meet two key requirements. First, the core process of salience computing (i.e., center-surround competition) had to be implemented at the neural level. Second, the model had to achieve eye-movement prediction performance comparable to that of established saliency map models used in previous eye-movement studies. We developed our model by extending the Brecht-Saiki model, which satisfies the first requirement but not the second, to ensure that both criteria were met. Our model incorporates an artificial neural network with excitatory center-inhibitory surround lateral connections (Figure 1), simulating the time course of neural population activity underlying center-surround competition (Figure 2). We demonstrated that its performance in predicting eye movements is comparable to that of the Itti-Koch model, a widely used model for eye-movement analysis, by setting balanced lateral connection parameters (Figure 3). We introduced disturbances into the neural network by modifying parameters, which resulted in changes in the computed salience difference between salient and non-salient objects. In other words, they altered the weighting of salience (Figure 4). Finally, we tested parameter estimation using artificially generated fixation data and demonstrated the potential application of our model in eye-movement analysis (Figure 5). These results suggest that individual differences in salience computing, particularly in terms of salience weighting, can be quantified by applying our model to eye-movement data.

Our findings indicate that salience must be appropriately weighted through balanced center-surround competition. This contrasts with a simple winner-take-all strategy, which detects only the most salient object and works even if salience for non-salient objects is completely eliminated. Neural networks with lateral connections are at risk of excessive competition owing to continuous and iterative interactions. One key mechanism to overcome this risk is the normalization of neural activity within a reasonable range. As in the Brecht-Saiki model, we introduced depressing effects (Equation 13) to simulate synaptic depression (Abbott et al., 1997), which helps regulate the activity of each neural population. The Brecht-Saiki model indicated that synaptic depression prevents overcompetition (de Brecht and Saiki, 2006), and our model similarly showed that steady-state activity for non-salient objects was not entirely eliminated (Figure 2). Our simulations with parameter modifications (Figure 4) suggest additional mechanisms for achieving balanced competition. Introducing excitatory-inhibitory imbalance, baseline neural activity, and reduced synaptic connections each affected lateral interactions and altered the weighting of salience. Notably, baseline neural activity did not modify lateral connections themselves but influenced salience weighting, likely owing to increased lateral signaling. Although the neural basis of abnormal visual salience remains unknown, our computational modeling in this study provides a hypothesis on how neurobiological characteristics influence visual salience. We showed that salience computing is normally well-balanced, but neurobiological abnormalities can cause unbalanced lateral interactions, thereby altering the weighting of salience.

The disturbances introduced into our model are relevant to neural mechanisms implicated in mental disorders. First, Yizhar et al. (2011) reported that excitatory-inhibitory imbalance in the mouse neocortex was associated with social dysfunction (Yizhar et al., 2011). Since excitatory glutamatergic pyramidal neurons and inhibitory GABAergic interneurons are indicated to play central roles in maintaining this balance, its disruption is linked to the glutamatergic hypothesis of schizophrenia (Uliana et al., 2024). Second, Jacob et al. showed that dopamine, a neurotransmitter implicated in mental disorders including schizophrenia, modulated the neural signal-to-noise ratio by altering the baseline activity of prefrontal neurons in monkeys (Jacob et al., 2013). Accordingly, changes in baseline activity can be associated with the dopaminergic hypothesis of schizophrenia (McCutcheon et al., 2020; Ott and Nieder, 2019). Finally, reduced synaptic connections are consistent to a study in which reduced dendritic spine density was observed in neocortical neurons of patients with schizophrenia in histological examinations (Garey et al., 1998).

The altered weighting of salience differs from the concept of abnormal salience described in conventional theory. The aberrant salience hypothesis, a theory of schizophrenia pathophysiology, suggests that the misattribution of motivational salience to stimuli irrelevant to external conditions may underlie schizophrenia symptoms (Kapur, 2003). Motivational salience is attributed based on reward prediction and is associated with reward-related learning, in which phasic changes in dopamine activity corresponding to reward prediction errors adjust corticostriatal learning to ensure accurate reward prediction (Bromberg-Martin et al., 2010). If phasic dopamine activity occurs independently of stimuli, motivational salience can become disturbed. Indeed, spontaneous phasic dopamine activity was observed in animals treated with amphetamine (Daberkow et al., 2013), which may lead to randomly assigned reward prediction and motivational salience unrelated to actual stimuli. In contrast to such random generation of salience, the altered weighting of salience proposed by our model remains stimuli-dependent. In this case, salience that should be moderate becomes excessively high or low (Figure 4). This pattern of alteration is also supported by empirical findings in eye-movement studies. Patients with schizophrenia tend to concentrate their gaze within a narrower area of the visual stimulus compared to healthy controls (Okada et al., 2021). Furthermore, the mean value of salience at fixation points, quantified using the Itti-Koch model, was higher in participants with schizophrenia than in healthy controls (Miura et al., 2025; Yoshida et al., 2024). These findings are consistent with our simulations, in which salience for non-salient object was attenuated by enhanced lateral interactions (Figure 4), and fixations artificially generated from saliency maps with such altered weighting tended to cluster in regions of high salience (Figure 5). Therefore, disturbances in salience processing may take different forms—some individuals may experience random salience generation, others may exhibit altered salience weighting, and some may be affected by both. To detect abnormalities in salience processing from experimental data, models that explicitly address how salience is disturbed are needed (Miyata et al., 2024).

Our computational modeling is based on simplified assumptions as we focused on bottom-up salience computing associated with excitatory center-inhibitory surround lateral interactions. In our model, signals corresponding to extracted features are sent to multiple maps within the artificial neural network and processed separately. However, the corresponding structures in the brain remain unclear, and these maps may not be spatially distinct in actual neural circuits. This issue relates to the binding problem (Treisman, 1996), which concerns how different types of feature information are processed separately and integrated. One proposed solution to the binding problem is temporal binding (Engel and Singer, 2001), which suggests that information integration and separation depends on whether neural activity synchronizes or not. Therefore, one possible extension of our model would be to introduce neural phase synchronization. Another approach is to separate information processing through more complex lateral connections. Although we did not consider neuron types in this study, the brain consists of various types of neurons, and synaptic connectivity depends on neuron type. A simple computational saliency map model proposed by Li includes pyramidal cells and interneurons in the primary visual cortex, where excitatory connections depend on whether neurons prefer similar orientations (Li, 2002). Information processing may be separable through connectivity patterns based on the specific features that presynaptic and postsynaptic neurons respond. Models incorporating various types of connectivity may account for additional bottom-up attentional mechanisms. A recent study on avian midbrain networks involved in salience computation indicated that bottom-up stimulus selection is modulated by local inhibitory surrounds that depend on stimulus feature similarity, in combination with global inhibitory surrounds that are independent of feature similarity (Qian et al., 2025). While incorporating such complex biological characteristics is beyond the scope of our current study, computational modeling based on detailed neurobiological findings may enable a more biologically realistic implementation of visual salience computation.

In this study, we considered the effect of altered bottom-up salience on eye movements; however, eye movements do not depend solely on bottom-up salience. They can also be influenced by top-down cognition (i.e., semantic understanding of scenes and goal-related information). In addition, the balance between bottom-up and top-down processes may be important for visual attention, as an eye-movement study suggested that schizophrenia patients tend to prioritize bottom-up visual salience over top-down cognition (Adámek et al., 2024). Evaluating abnormalities in bottom-up visual salience is still possible by applying saliency map models to eye-movement data (Miura et al., 2025; Yoshida et al., 2024) because bottom-up salience computation itself may not be disturbed by top-down cognition, and eye movements reflect the effect of bottom-up salience, which can be extracted using saliency map models.

For more precise analyses of abnormal salience, however, we face the challenge of distinguishing altered bottom-up salience, altered top-down cognition, and an imbalance between bottom-up and top-down processes. To address this issue, it is important to consider that top-down cognition depends heavily on whether visual stimuli are interpretable. For example, top-down cognition would have strong effects on eye movements for meaningful stimuli and weak effects for meaningless stimuli. Measuring eye movements with various types of visual stimuli is therefore useful, as demonstrated by the CAT2000 dataset used in this study, which includes 20 categories of images (Borji and Itti, 2015). It is also notable that the effects of top-down processes do not appear immediately after stimulus presentation but occur later in time (Theeuwes, 2010). Data acquisition and analysis based on stimulus type and time after presentation may help distinguish top-down and bottom-up factors. Using computational models that incorporate top-down cognition in combination with models designed solely for bottom-up salience is also beneficial. Deep learning-based saliency map models are typically trained in an end-to-end manner to predict eye movements directly from visual stimuli, and are thus considered to reflect both bottom-up salience and top-down cognition. This characteristic may explain their high performance in eye-movement prediction. Indeed, such models outperform those designed only to capture bottom-up salience. For example, Kroner et al. evaluated their proposed deep learning-based model using the same dataset and the metrics (CAT2000 and NSS) as those employed in the present study, reporting an NSS score of 2.30 (Kroner et al., 2020)—substantially higher than the scores achieved by bottom-up salience models, including ours, which reached at most around 1.15, as shown in Figure 3. However, simply applying such deep learning-based models to data analysis makes it difficult to disentangle bottom-up and top-down effects on eye movements. As demonstrated by Adámek et al., analyzing eye movements with both bottom-up and deep learning-based models, and comparing their results, enables a more distinct evaluation of these two types of influences (Adámek et al., 2024). Taken together, our proposed model has the potential to quantify altered weighting of salience as reflected in eye movements, and this potential can be further enhanced through refined experimental designs and analyses.

## Data Availability

The data supporting the findings of this study were obtained through simulations based on the computational model, as well as derived from model-driven analyses applied to the publicly available CAT2000 dataset. The MATLAB codes used for the model and simulations are available on GitHub at https://github.com/fujitaysim/saliency_map. The CAT2000 dataset can be accessed at https://saliency.tuebingen.ai/datasets.html.
